# Effects of whole-body vibration training with different frequencies on the balance ability of the older adults: a network meta-analysis

**DOI:** 10.3389/fphys.2023.1153163

**Published:** 2023-04-17

**Authors:** Peirong Liu, Yongjie Li, Yajun Xiao, Duo Li, Lin Liu, Yong Ma, Weitao Zheng

**Affiliations:** ^1^ Key Laboratory of Sports Engineering of General Administration of Sports of China, Research Center of Sports Equipment Engineering Technology of Hubei Province, Wuhan Sports University, Wuhan, China; ^2^ Department of Rehabilitation Medicine, Beijing Jishuitan Hospital Guizhou Hospital, Guiyang, China

**Keywords:** whole body vibration, frequency, older adults, balance, meta-analysis

## Abstract

**Purpose:** To compare the effects of whole-body vibration training (WBVT) with different frequencies on the balance ability of older adults.

**Methods:** Randomized controlled trials (RCTs) on the WBVT interventions on balance ability in older adults were searched through PubMed, Web of Science, The Cochrane Library, ProQuest, Embase, Opengrey, China National Knowledge Infrastructure (CNKI), Wanfang, and China Science and Technology Journal Database (CSTJ) databases from the establishment of the database to August 2022, and all literature that met the PICOS (Participants, Intervention, Comparison, Outcomes, Study design) criteria were enrolled. Two reviewers screened and assessed the methodological quality of the included literature according to the physiotherapy evidence database (PEDro) scale criteria. Statistical analysis was performed using Stata 14.0 software after data extraction.

**Results:** Twenty-five RCTs with a total of 1267 subjects were finally included. The results of the pairwise comparison of the Network Meta-analysis showed that the Timed Up and Go Test (TUGT) values of Low-frequency whole-body vibration training (LF-WBVT) was lower than the placebo and traditional rehabilitation groups, and the difference was statistically significant [WMD = −1.37, 95% CI (−2.53, −0.20)] [WMD = −1.84, 95% CI(-3.17,-0.51)]. The Five-repetition Sit-to-Stand Test (5STS) values of LF-WBVT, Medium-frequency whole-body vibration training (MF-WBVT), and High-frequency whole-body vibration training (HF-WBVT) were lower than the placebo and traditional rehabilitation groups, but none of them were statistically significant. In addition, the TUGT and 5STS values of HF-WBVT had a tendency to be lower than those of LF-WBVT and MF-WBVT, but neither of them was statistically different. The cumulative probability ranking results of both TUGT and 5STS showed that HF-WBVT was the best protocol.

**Conclusion:** Current evidence shows that HF-WBVT may be the best protocol for improving balance in older adults. Due to the study’s limitations, the conclusion obtained in this study still needs to be further confirmed by more high-quality studies.

**Systematic Review Registration**: [https://www.crd.york.ac.uk/PROSPERO/], identifier [CRD42021250405].

## 1 Introduction

Falls are one of the leading causes of disability, injury, and death in older adults and are a significant public health problem worldwide (Montero-Odasso, et al., 2022). Studies have shown that at least 30% of people over 65 experience falls yearly, and this percentage increases to 50% for those over 80 (Montero-Odasso, et al., 2021). Falls can have serious consequences, such as pain, reduced function, limited self-care, and even fractures (Gates, et al., 2008; Gillespie, et al., 2012; Burns, et al., 2016). In addition, falls can lead to severe psychological impairment, fear, and loss of confidence, resulting in reduced physical activity and social interaction in older adults, further reducing mobility and increasing the risk of recurrent falls (Sherrington, et al., 2019). In the elderly, reduced balance and postural control with increasing age have been identified as significant risk factors for falls (Lord, et al., 1992; Ganz and Latham, 2020). Early development of appropriate exercise intervention programs for older adults can reduce the risk of falls (Morley, et al., 2013).

Traditional resistance exercises and balance training can effectively improve physical fitness and reduce the chances of falls in older adults (von Stengel, et al., 2012). However, to obtain significant improvements in balance and other physical functions, the total dose of exercise must be of considerable intensity and sufficiently long duration (Sherrington, et al., 2020). Thus, older adults sometimes feel very fatigued and thus need more motivation (Burton, et al., 2017). In addition, other age-related limitations, such as cardiac limitations, muscle strength limitations, or lack of relevant training conditions, such as space and time, will reduce the feasibility of the training program (Goudarzian, et al., 2017).

Whole-body vibration training (WBVT) generates vibrations through a vibrating platform that produces shock stimuli to body muscle groups, which can increase active muscle activity, improve the biological activity of high threshold motor units and increase neuromuscular excitability (Bernardo-Filho, et al., 2018; Wuestefeld, et al., 2020; van Heuvelen, et al., 2021). It has been demonstrated that WBVT can improve balance and enhance body control in older adults, thereby reducing the incidence of falls (Jepsen, et al., 2017), and compared with traditional training, WBVT reduces stress on the musculoskeletal, cardiovascular, and respiratory systems (Bogaerts, et al., 2009), making it safer and more acceptable for older adults with relatively mild side effects (Kawanabe, et al., 2007; Gomes-Neto, et al., 2019; Tseng, et al., 2021; Liu, et al., 2022). Relevant Meta-analyses have been published and confirmed the method’s effectiveness (Rogan, et al., 2011; Lam, et al., 2012; Orr, 2015; Rogan, et al., 2017). Frequency, a key parameter of WBVT, is necessary for successful fall risk reduction and can determine its effectiveness of WBVT and outcome (Sherrington, et al., 2008; Dutra, et al., 2016; van Heuvelen, et al., 2021). However, current evidence has been obtained by comparing each experimental group with the control group at the same vibration frequency, and valid comparisons between different frequencies still need to be made. Therefore, this study will use a Network Meta-analysis to compare the effects of WBVT with different vibration frequencies on the balance ability of older adults to provide practical, evidence-based clinical support.

## 2 Materials and methods

The systematic review was performed according to the Preferred Reporting Items for Systematic Reviews and Meta-Analysis (PRISMA) guidelines (Page, et al., 2021). This study has been registered with the International Prospective Registry of Systematic Evaluations (PROSPERO) under the registration number CRD42021250405.

### 2.1 Eligibility criteria

#### 2.1.1 Inclusion criteria

The inclusion criteria were set based on participants, intervention, comparison, outcomes, and study design (PICOS) strategy. Participants (P): Healthy older adults without chronic diseases, neuromusculoskeletal systems, and joint injuries who had not undergone high-intensity physical training 6 months before the trial. Intervention (I): Received one of the interventions which included LF-WBVT (<30 Hz), MF-WBVT (≥30Hz, <45 Hz), and HF-WBVT (>45 Hz) (Yeung and Yeung, 2015; Wei, et al., 2017; Sajadi, et al., 2019). Comparison (C): The placebo comparison included no intervention and sham intervention, of which the latter referred to the analog vibration without any effective vibration stimulation. Traditional rehabilitation, such as strength training, physical therapy, and usual care, was acceptable as cointervention. Outcomes (O): Primary outcome measures in this study assessed the balance ability, including the Timed Up and Go Test (TUGT) and the Five-repetition Sit-to-Stand Test (5STS). TUGT and 5STS are clinically used to measure balance ability (Podsiadlo and Richardson, 1991; Whitney, et al., 2005; Hwang, et al., 2018; Phu, et al., 2019). Study design (S): Randomized controlled trials (RCTs).

#### 2.1.2 Exclusion criteria

The exclusion criteria adopted in the present study are as follows: 1) reviews, abstracts, and conference reports; 2) lack of outcome measures; 3) multiple publications of the literature; 4) unavailability of full text; 5) concurrent medication therapy during the intervention.

### 2.2 Operational definitions

WBVT was defined as an exercise intervention, consisting of the application of an oscillation platform that transmits mechanical stimuli to the human body standing on it (de Oliveira, et al., 2023).

### 2.3 Databases and search strategies

A systematic search was conducted through PubMed, Web of Science, The Cochrane Library, ProQuest, Embase, Opengrey, CNKI, Wanfang, and CSTJ databases on randomized controlled trials of WBVT interventions on balance ability in older adults, and the search time was controlled from the establishment of the database to August 2022.

The search terms include balance, fall, old people, vibration, and whole-body vibration training. The specific search formula (PubMed was used as an example): (balance OR fall OR equilibrium) AND (older adults OR older people OR aging OR elder OR old) AND (vibration OR whole-body vibration training OR WBVT). A second search was conducted for the list of references and citations included in the study to identify other possible relevant studies.

The PICOS strategy (Costantino, et al., 2015) was used to structure the bibliographic search: Participants (P) = older adults; Intervention (I) = WBVT; Comparison (C) = placebo or traditional rehabilitation; O (outcome) = TUGT; 5STS. Study design (S): RCTs.

### 2.4 Study selection and data extraction

Two reviewers screened the entire retrieved literature. The EndnoteX9 software was used in the first step to delete duplicate data. The titles and abstracts were read according to the previously set inclusion criteria, leaving those that met the requirements. The full text was read later to determine the final inclusion. For any disagreement, the studies will be re-evaluated. If the disagreement cannot be resolved, a group discussion will be held to resolve it.

In the second step, Information related to the included studies was extracted and collected into an Excel sheet. The extracted Information included: the first author’s name, country of publication, year of publication, sample size (experimental and control groups), age, interventions (experimental and control groups), outcome indicators, frequency of intervention, and duration of intervention.

### 2.5 Methodological quality and risk of bias

Two reviewers evaluated the methodological quality of the included studies according to the criteria of the Physiotherapy Evidence Database (PEDro) scale (Cashin and McAuley, 2020). It consists of 11 items with a total score of 10 (the final score does not include the “inclusion criteria” items). A PEDro score of ≥6 indicates a high methodological quality for an included study. The Cochrane Collaboration’s tool was used to assess the risk of bias of the included studies (Higgins, et al., 2011). The main areas of assessment are randomization, allocation concealment, blinding of participants and personnel, blinding of outcome assessment, completeness of data regarding outcomes, selective reporting, and other bias. When the evaluation was completed, the two reviewers exchanged and compared the results of both evaluations. The study team will decide through a group discussion if there is any disagreement.

### 2.6 Statistical analysis

#### 2.6.1 Network meta-analysis

Network Meta-analysis and graphical plotting were performed for relevant data using Stata 16.0 software. Since the outcome indicators in this study were all continuous variables and were evaluated by the same scale, the weighted mean difference (WMD) and 95% confidence interval (CI) were used as effect sizes. Network evidence plots for direct comparisons among interventions were first drawn; then, the consistency of the closed loop of each outcome indicator was evaluated by the ring inconsistency test, and when the 95% CI of the ring inconsistency factor (IF) contained 0, it indicated a good agreement between direct and indirect evidence. The results of the Network Meta-analysis were presented by pairwise comparison of forest plots. Cumulative ranking probability plots were drawn based on the surface under the cumulative ranking curve (SUCRA) to determine the optimal training frequency.

#### 2.6.2 Publication bias

We evaluated the publication bias of these studies and minor sample effects by visually measuring the symmetry of funnel plots in Stata16.0 software.

## 3 Results

### 3.1 Search results

**FIGURE 1:**
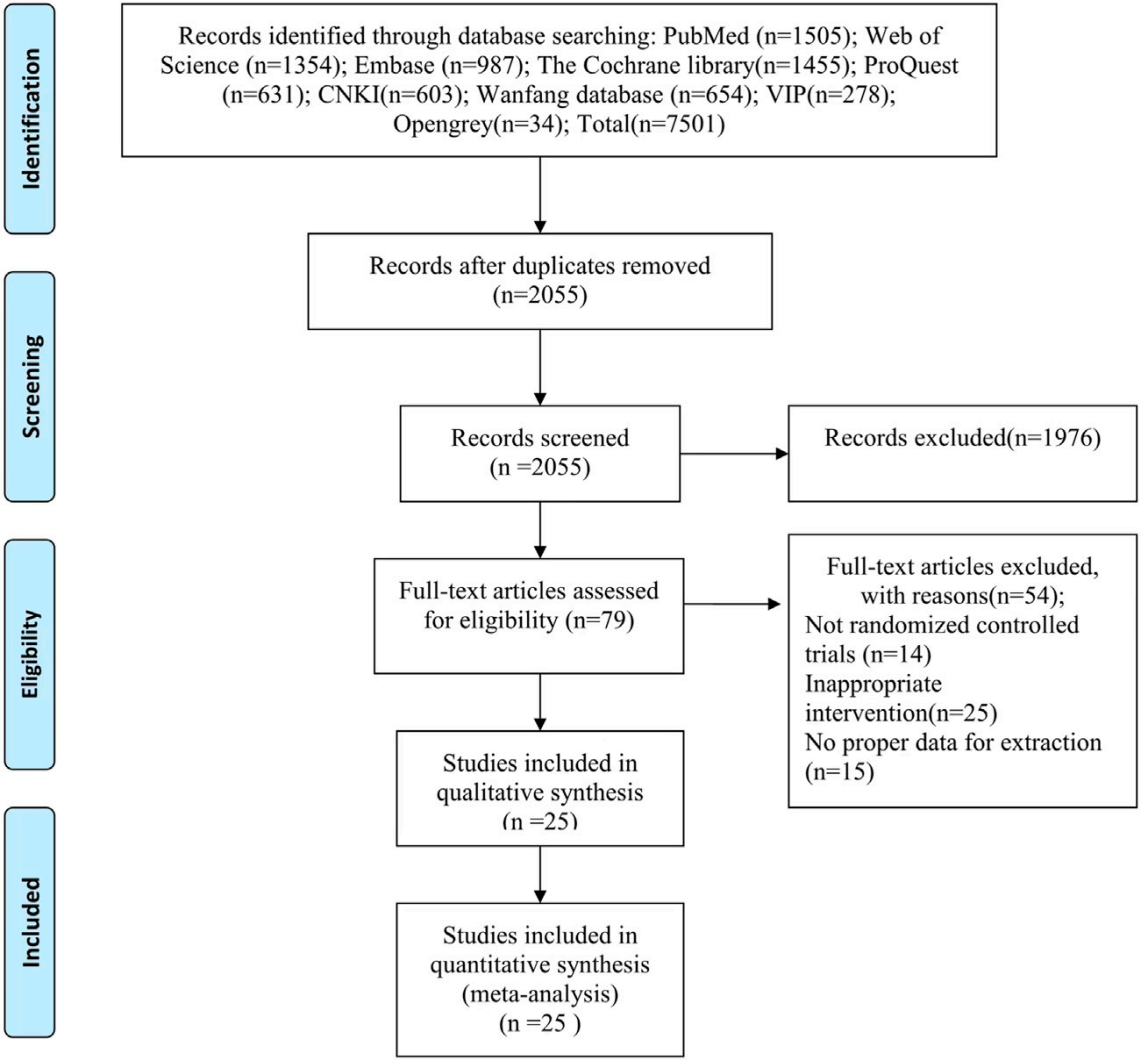
Flowchart of the study selection process.

A total of 7501 studies was retrieved, and 25 was finally included after multiple screenings (Bautmans, et al., 2005; Bruyere, et al., 2005; Rees, et al., 2007; Furness and Maschette, 2009; Furness, et al., 2010; Marín, et al., 2011; Pollock, et al., 2012; Calder, et al., 2013; Buckinx, et al., 2014; Zhang, et al., 2014; Álvarez-Barbosa, et al., 2014; Corrie, et al., 2015; Ochi, et al., 2015; Santin-Medeiros, et al., 2015; Sitjà-Rabert, et al., 2015; Sucuoglu, et al., 2015; Lei, et al., 2016; Goudarzian, et al., 2017; Wei, et al., 2017; Lam, et al., 2018; Jingwang, et al., 2019; Zhu, et al., 2019; Sen, et al., 2020; Wadsworth and Lark, 2020; Xiao-feng, 2020). A flowchart of the study selection process is shown in Figure 1. The basic characteristics of included studies are shown in Table 1.

**TABLE 1:** The basic characteristics of included studies.

Study (author/year)	Country	Sample size (EG/CG)	Age (years old)	Interventions (EG/CG)	WBVT frequency (Hz)	WBVT amplitude (mm)	Duration of intervention (weeks)	Main outcomes
Álvarez-Barbosa, et al. (2014)	Spain	22 (11/11)	84.0 ± 3.0	MF-WBVT/Traditional rehabilitation	30–35	4	8	**①②**
Bautmans, et al. (2005)	Belgium	21 (10/11)	77.5 ± 11.0	MF-WBVT/Traditional rehabilitation	30–40	2–5	6	**①**
Bruyere, et al. (2005)	Belgium	36 (16/20)	81.9 ± 6.9	LF-WBVT/Traditional rehabilitation	10, 26	3, 7	6	**①**
Buckinx, et al. (2014)	Belgium	62 (31/31)	83.2 ± 7.9	MF-WBVT/Placebo	30	2	24	**①**
Calder, et al. (2013)	New Zealand	38 (19/19)	80.1	LF-WBVT/Traditional rehabilitation	20	2	6	**①②**
Corrie, et al. (2015)	United Kingdom	61 (21/20)	80.2 ± 6.5	LF-WBVT/Traditional rehabilitation	28.4, 29.8	1.3, 2.9	12	**①②**
Furness and Maschette (2009)	Australia	37 (19/18)	72.0 ± 8.0	LF-WBVT/Placebo	15–25	0.05	6	**①②**
Furness, et al. (2010)	Australia	37 (19/18)	69.0 ± 8.0	LF-WBVT/Placebo	15–25	1	12	**①②**
Goudarzian, et al. (2017)	Iran	20 (11/9)	68.0 ± 5.8	MF-WBVT/Placebo	30–35	5–8	8	**①②**
Lam, et al. (2018)	China	62 (21/19/22)	82.3 ± 7.3	MF-WBVT/Traditional rehabilitation/Placebo	30–40	0.9	8	**①②**
Marín, et al. (2011)	Spain	60 (11/11)	84.3 ± 7.4	MF-WBVT/Placebo	30–40	1.05–2.11	8	**②**
Ochi, et al. (2015)	Japan	20 (10/10)	80.9 ± 2.8	LF-WBVT/Traditional rehabilitation	10–21	3–7	12	**①**
Pollock, et al. (2012)	United Kingdom	77 (38/39)	80.0 ± 1.4	LF-WBVT/Traditional rehabilitation	15–30	2–8	8	**①**
Rees, et al. (2007)	Australia	28 (15/13)	74.3 ± 5.0	LF-WBVT/Traditional rehabilitation	26	5–8	32	**①②**
Santin-Medeiros, et al. (2015)	Spain	27 (19/18)	82.4 ± 5.7	LF-WBVT/Placebo	20	2	32	**①②**
Sen, et al. (2020)	Turkey	33 (15/16/18)	55.0 ± 4.6	MF-WBVT/Traditional rehabilitation/Placebo	30–40	2–4	12	**①**
Sitjà-Rabert, et al. (2015)	Spain	159 (81/78)	82.0	MF-WBVT/Traditional rehabilitation	30–35	2–4	6	**①②**
Sucuoglu, et al. (2015)	Turkey	42 (21/21)	58.76 ± 5.82	WBVT/Traditional rehabilitation	30–35		4	**①**
Wadsworth and Lark (2020)	New Zealand	55 (27/30/28)	82.45 ± 7.9	LF-WBVT/Traditional rehabilitation/Placebo	6–26	2–4	16	**①**
Wei, et al. (2017)	China	120 (20/20/20/20)	78.0 ± 4.0	HF-WBVT/MF-WBVT/LF-WBVT/Placebo	60,40,20	4	12	**①②**
Zhang, et al. (2014)	China	37 (19/18)	85.27 ± 3.63	LF-WBVT/Traditional rehabilitation	6–26	1–3	8	**①②**
Zhu, et al. (2019)	China	55 (28/27)	87.5 ± 3.0	LF-WBVT/Placebo	12–16	3–5	8	**①②**
Xiao-feng (2020)	China	80 (40/40)	64.75 ± 2.45	HF-WBVT/MF-WBVT	30, 45	2	12	**①②**
Lei, et al. (2016)	China	42 (22/20)	72.73 ± 1.46	MF-WBVT/Traditional rehabilitation	35–45	5	8	**①**
Jingwang, et al. (2019)	China	36 (20/16)	84.6 ± 5.7	LF-WBVT/Traditional rehabilitation	3–13	3	12	**①②**

Note: LF-WBVT, Low-frequency whole-body vibration training; MF-WBVT, Medium frequency whole-body vibration training; HF-WBVT, High-frequency whole-body vibration training; ① = Timed Up and Go Test (TUGT); ② = Five-repetition Sit-to-Stand Test (5STS).

### 3.2 Quality evaluation

Table 2 shows the results of the methodological quality evaluation of the included studies. The scores of the included studies ranged from 5 to 8, with 18 having a PEDro score of 6 or more. All studies described inclusion criteria and underwent random assignment, and 5 studies underwent allocation concealment; all studies were comparable at baseline; 3 studies were blinded to subjects, no studies were blinded to therapists, and 13 studies were blinded to assessors; all studies had a clinical sample dropout rate of less than 15%; 16 studies used intentional analysis; all included studies underwent between-group statistics, point measures, and difference value statistics.

**TABLE 2:** Physiotherapy evidence database (PEDro) scores of the included studies.

Study	1	2	3	4	5	6	7	8	9	10	11	PEDro score
Álvarez-Barbosa, et al. (2014)	●	●	○	●	○	○	○	●	●	●	●	6
Bautmans, et al. (2005)	●	●	●	●	○	○	●	●	●	●	●	8
Bruyere, et al. (2005)	●	●	○	●	○	○	○	●	●	●	●	6
Buckinx, et al. (2014)	●	●	○	●	○	○	●	●	●	●	●	7
Calder, et al. (2013)	●	●	○	●	○	○	●	●	○	●	●	6
Corrie, et al. (2015)	●	●	○	●	●	○	●	●	○	●	●	7
Furness and Maschette (2009)	●	●	○	●	○	○	○	●	○	●	●	5
Furness, et al. (2010)	●	●	○	●	○	○	○	●	○	●	●	5
Goudarzian, et al. (2017)	●	●	○	●	○	○	○	●	○	●	●	5
Lam, et al. (2018)	●	●	●	●	●	○	●	●	●	●	●	9
Marín, et al. (2011)	●	●	○	●	○	○	○	●	○	●	●	5
Ochi, et al. (2015)	●	●	●	●	○	○	●	●	●	●	●	8
Pollock, et al. (2012)	●	●	●	●	●	○	●	●	●	●	●	9
Rees, et al. (2007)	●	●	○	●	○	○	○	●	○	●	●	5
Santin-Medeiros, et al. (2015)	●	●	○	●	○	○	○	●	●	●	●	6
Sen, et al. (2020)	●	●	○	●	○	○	○	●	●	●	●	6
Sitjà-Rabert, et al. (2015)	●	●	●	●	○	○	●	●	●	●	●	8
Sucuoglu, et al. (2015)	●	●	○	●	○	○	●	●	○	●	●	6
Wadsworth and Lark (2020)	●	●	○	●	○	○	○	●	●	●	●	6
Wei, et al. (2017)	●	●	○	●	○	○	●	●	●	●	●	7
Zhang, et al. (2014)	●	●	○	●	○	○	●	●	●	●	●	7
Zhu, et al. (2019)	●	●	○	●	○	○	○	●	●	●	●	6
Xiao-feng (2020)	●	●	○	●	○	○	○	●	○	●	●	5
Lei, et al. (2016)	●	●	○	●	○	○	●	●	●	●	●	7
Jingwang, et al. (2019)	●	●	○	●	○	○	●	●	●	●	●	7

Note: 1 = Eligibility criteria; 2 = Randomized Allocation; 3 = Blinded Allocation; 4 = Group homogeneity; 5 = Blinded subjects; 6 = Blinded therapists; 7 = Blinded assessor; 8 = Drop out <15%; 9 = Intention to treat analysis; 10 = Between group comparison; 11 = Point estimates and variability. ● adds a point to the score, ○ adds no point to the score. The item “eligibility criteria” is not included in the final score.

**FIGURE 2:**
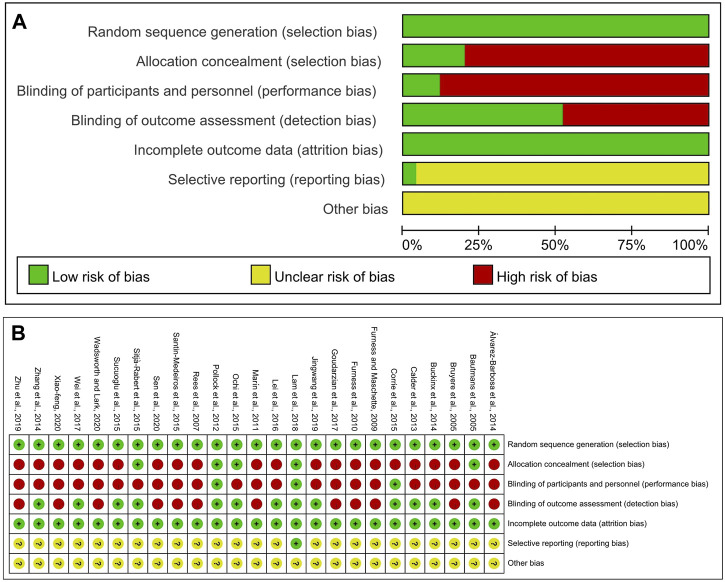
Rias of bias assessment results. **(A)** Risk of bias graph; **(B)** Risk of bias summary.

Figure 2 shows the results of the risk of bias in included studies. All studies reported the specific random mode, and 5 studies revealed the detailed allocation concealment; 3 studies were blinded to participants and personnel; 13 studies were blinded to assessors. Regarding the completeness of data regarding outcomes, all studies were rated as having a low risk of bias. Only 1 study showed a small probability of selection bias, and the remaining studies had an unclear risk of bias because they did not provide sufficient information to rule out bias.

### 3.3 Network meta-analysis

#### 3.3.1 TUGT

**FIGURE 3:**
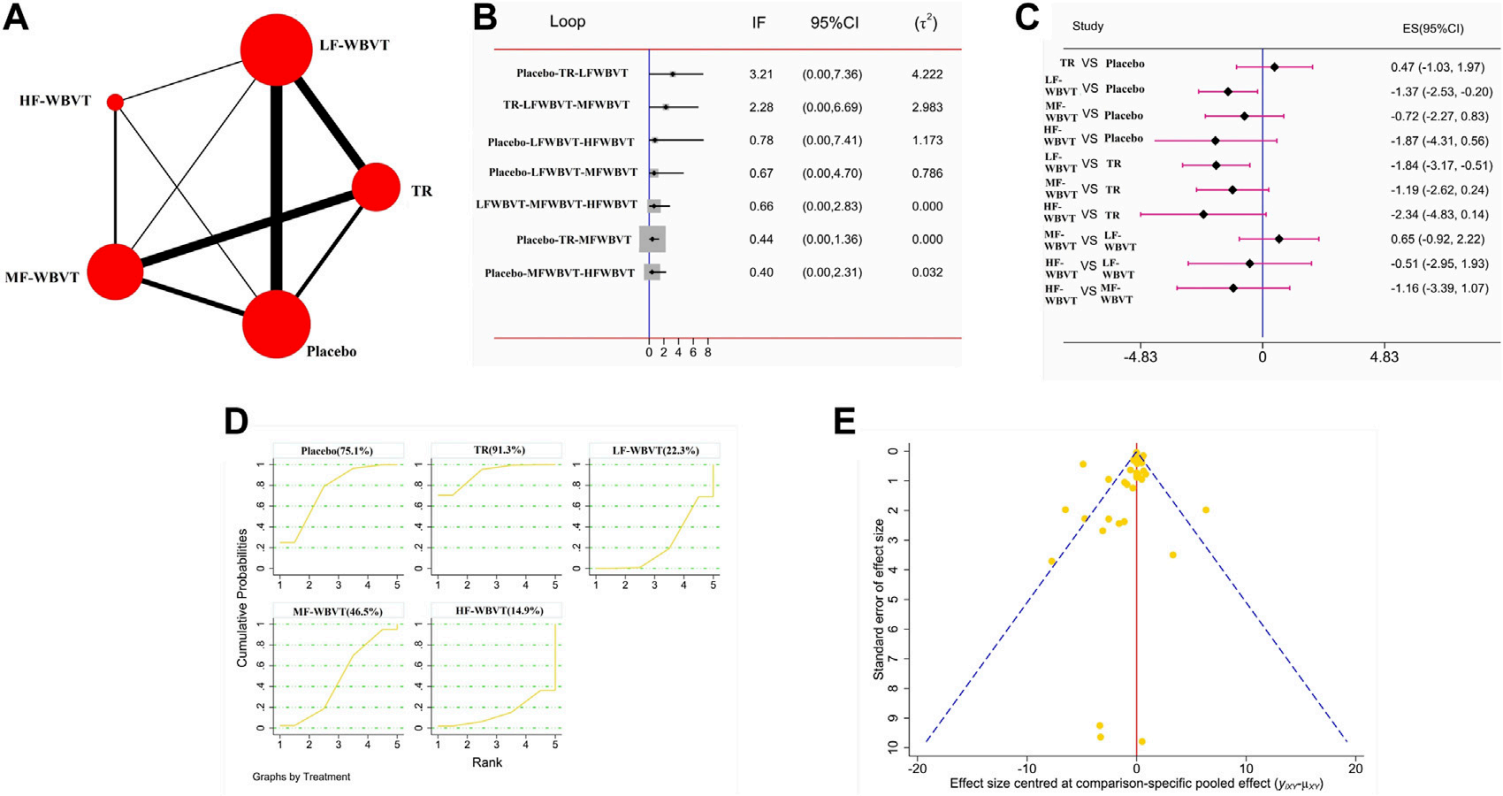
Network meta-analysis results for TUGT. **(A)** Network evidence diagram; **(B)** loop inconsistency test; **(C)** Forest plot; **(D)** The figure of Cumulative probability ranking; **(E)** funnel plot.

A total of 24 studies and 1207 subjects was included. The network evidence diagram is shown in Figure 3A. The results of the loop inconsistency test manifested that the 95% CI of the loop inconsistency factor (IF) contained 0, indicating good consistency (Figure 3B). The forest plot of pairwise comparison (Figure 3C) showed that the TUGT values were lower in LF-WBVT [WMD = −1.37,95% CI (−2.53,−0.20)] [WMD = −1.84,95% CI (−3.17,−0.51)] compared to the placebo and traditional rehabilitation groups, with statistically significant differences; MF-WBVT and HF WBVT had lower TUGT values than the placebo and traditional rehabilitation groups, but the differences were not statistically significant. In addition, TUGT values were lower in HF-WBVT compared to LF-WBVT and MF-WBVT, but the difference was not statistically significant. The cumulative probability ranking results (Figure 3D) showed a SUCRA value of 14.9% for HF-WBVT, indicating that HF-WBVT may be the best option.

#### 3.3.2 5STS

**FIGURE 4:**
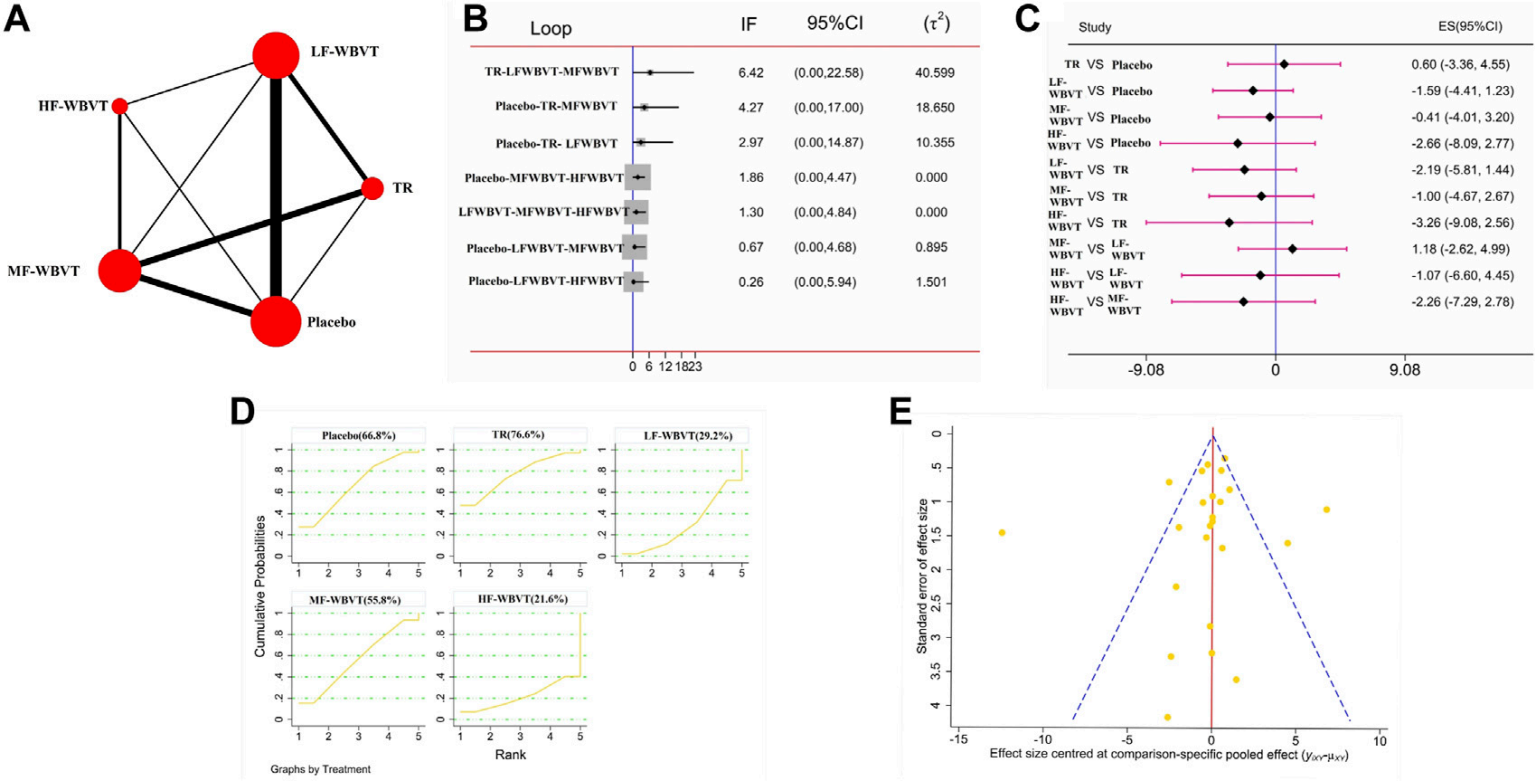
Network meta-analysis results for 5STS. **(A)** Network evidence diagram; **(B)** loop inconsistency test; **(C)** Forest plot; **(D)** The figure of Cumulative probability ranking; **(E)** funnel plot.

A total of 16 studies and 879 subjects was included. The network evidence diagram is shown in Figure 4A. The results of the loop inconsistency test manifested that the 95% CI of the loop inconsistency factor (IF) contained 0, indicating good consistency (Figure 4B). The forest plot of pairwise comparison (Figure 4C) showed that the 5STS values were lower for LF-WBVT, MF-WBVT, and HF-WBVT compared to the placebo and traditional rehabilitation groups, but none of them were statistically significant. In addition, the 5STS values were lower in HF-WBVT compared to LF-WBVT and MF-WBVT, but the difference was not statistically significant. The cumulative probability ranking results (Figure 4D) showed a SUCRA value of 21.6% for HF-WBVT, indicating that HF-WBVT may be the best option.

### 3.4 Publication bias

As seen in Figure 3E and Figure 4E, the funnel plot of the two indicators was symmetrical, and most of the points were in the upper part of the funnel. Although some of the few points fell outside the funnel plot, the overall results indicate that publication bias was less likely in this study, but the results need to be interpreted with caution.

## 4 Discussion

To the best of our knowledge, this is the first Network Meta-analysis study exploring the effect of different frequencies of WBVT on balance ability in older adults. The results showed that the SUCRA values of HF-WBVT were 14.9% and 21.6% in TUGT and 5STS, respectively, suggesting that HF-WBVT was the best WBVT protocol.

Previous studies have shown that TUGT and 5STS have good reliability and validity in assessing balance (Goldberg, et al., 2012; Paineiras-Domingos, et al., 2018; Phu, et al., 2019; Nawrat-Szołtysik, et al., 2022). In the present study, the effect of WBVT on balance in older adults was assessed by TUGT and 5STS. The pairwise comparison results showed that the overall majority of the frequency groups had no statistically significant TUGT and 5STS values compared to the placebo and traditional rehabilitation groups but had a tendency to decrease. This improvement may be related to the WBVT-induced tonic vibratory reflex. The vibratory waves induced by the vibration platform stimulate proprioceptors, increase the excitability of afferent fibers, activate α motor neurons, and cause reflex muscle contractions, thus improving neuromuscular performance and balance in older adults (Rogan, et al., 2017; Bemben, et al., 2018). In addition, during WBVT application, the active and synergistic muscles produce muscular contractions, causing changes in the length of the muscle shuttle and activating the tendon apparatus, resulting in a timely relaxation of the antagonist’s muscles, thus increasing the efficiency of muscle contraction, improving the coordination of muscle activity and making it more precise (Ritzmann, et al., 2018). These neural adaptations play a crucial role in the balance and movement of the body.

In addition, the pairwise comparison of each frequency group showed that the TUGT and 5STS values of HF-WBVT tended to be lower than those of MF-WBVT and HF-WBVT. The cumulative probability ranking results also showed that HF-WBVT had the smallest SUCRA values in TUGT and 5STS, suggesting that HF-WBVT may be the best protocol. One possible reason is that neuromuscular tissues resonate under high-frequency vibration stimulation. Vibration frequency is defined in Hertz (Hz) and indicates the number of cycles per second (Rauch, et al., 2010; van Heuvelen, et al., 2021). Within a specific range, the higher the vibration frequency, the faster the intramuscular fiber length of the muscle spindle changes, and the frequency and intensity of nerve-issued impulses increase, triggering a more intense tonic vibration reflex and recruiting more motor units to participate in movement, which increases the level of neuromuscular activation and ultimately leads to improved balance. A previous study has reported that the electromyography (EMG) responses of subjects observed by EMG at different frequencies of WBVT were found to be more active at HF-WBVT, indicating that HF-WBVT triggered more intense muscle activity (Hazell, et al., 2007). Furthermore, HF-WBVT may increase the gravitational loading exerted on the neuromuscular system, thereby improving neuromuscular performance. It has been suggested that WBVT activates muscles through vibration to promote strength and motor performance, a mechanism similar to the neuromuscular effects achieved by traditional resistance training (Delecluse, et al., 2003; Roelants, et al., 2004; Bogaerts, et al., 2007), an exercise method that uses external resistance (such as dumbbells, barbells, elastic bands, etc.) to enhance muscle strength and physical fitness (Zuo, et al., 2022). A previous animal experimental study applied several frequencies and found that the higher the frequency, the better the effect of muscle strength improvement (Lin, et al., 2015). Similarly, the stronger effects of higher frequencies are in agreement with the results findings of reviews of chronic obstructive pulmonary disease (COPD) patients and Parkinson’s disease (PD) patients and can be explained by enhanced neuromuscular activity (Ritzmann, et al., 2018; Berner, et al., 2020; Laurisa, et al., 2022).

It is worth mentioning that, due to the delivery of vibration stimuli from the lower extremities from bottom to top, the damping of the vibration waves through the joints, muscles, and soft tissues leads to a certain degree of attenuation of the stimuli (Luo, et al., 2005; Oroszi, et al., 2020). Lower frequencies may not transmit the vibration stimuli effectively, while higher frequencies will more frequently and better enable the transmission of vibration stimuli and influence the neuromuscular response. Lower (less than 20 Hz) vibration frequencies may not be effective because organs in the body vibrate at similar frequencies (Dincher, et al., 2021). In addition, no adverse effects were reported in any of the studies. However, vibration stimulation of too long a duration may have adverse effects (Cardinale and Wakeling, 2005). Some studies reported that prolonged exposure to HF-WBVT shifts the nerve impulses released by Ia from enhancement to inhibition and predisposes the central nervous system (CNS) to fatigue and protective inhibition (Bongiovanni, et al., 1990; Da Silva-Grigoletto, et al., 2011; Lu, et al., 2021). Therefore, the intervention duration of HF-WBVT still needs further study.

### 4.1 Strengths

The strengths of this study include that the evidence was obtained from RCTs, followed the PRISMA guidelines of reporting, and registered at PROSPERO to improve transparency. The most effective application frequency of WBVT to improve the balance ability of healthy older adults was determined through Network meta-analysis, providing a valuable reference for clinical application.

### 4.2 Study limitations

This study also had certain limitations, 1) the methodological quality and intervention duration of the included studies were not entirely consistent, which may bring potential heterogeneity; 2) only Chinese and English studies were included, which may have language bias; 3) the studies related to HF-WBVT was less included, which may affect the validity of the test and weaken the demonstration to a certain extent.

## 5 Conclusion

In conclusion, HF-WBVT may be the best protocol to improve balance in older adults. Due to the study’s limitations, the conclusions obtained in this study still need to be further confirmed by more high-quality, standardized, and extensive sample-size studies.

## Data Availability

The original contributions presented in the study are included in the article/supplementary material, further inquiries can be directed to the corresponding author.
